# Association of metabolic evaluation of visceral fat score with nonalcoholic fatty liver disease and liver fibrosis: A cross-sectional study based on NHANES

**DOI:** 10.1097/MD.0000000000042213

**Published:** 2025-04-25

**Authors:** Xiaomei Xu, Junping Yang, Yuanyuan Li, Yang Li, Xiaoyan Zeng, Biquan Chen

**Affiliations:** a Department of Infectious Diseases, Anhui Provincial Children’s Hospital, Hefei, Anhui, China; b Department of General Practice, Wuhu Second People’s Hospital, Wuhu, China.

**Keywords:** cross-sectional study, liver fibrosis, METS-VF index, NHANES, nonalcoholic fatty liver disease, obesity

## Abstract

The aim of this study was to investigate the correlation between the metabolic evaluation of visceral fat score (METS-VF) with nonalcoholic fatty liver disease (NAFLD) and liver fibrosis within an adolescent population. A cross-sectional analysis of data from the National Health and Nutrition Examination Survey (NHANES) involving 1274 subjects was conducted. Multiple linear regression was employed to ascertain the association between METS-VF with NAFLD and liver fibrosis. Smoothed curve fitting and threshold effect models were utilized to explore potential nonlinear relationships. Subgroup analyses were conducted to assess the stability of the relationship across different demographic groups. METS-VF exhibited a positive association with both NAFLD and liver fibrosis. In models adjusted for all covariates, the odd ratios (ORs) for METS-VF with NAFLD and liver fibrosis were 15.74 (95% CI: 10.44–23.72) and 1.85 (95% CI: 1.27–2.70), respectively. This positive correlation strengthened with increasing METS-VF when expressed in tertiles (*P*-value for trend < 0.01). Smoothed curve fitting and threshold effect analysis revealed a nonlinear correlation between METS-VF and NAFLD (log-likelihood ratio [LLR] < 0.01), with a more significant positive correlation observed when METS-VF exceeded 5.75 (OR = 104.42, 95% CI: 17.40–626.58). Similarly, a nonlinear correlation was observed between METS-VF and liver fibrosis (LLR < 0.01), with a stronger positive correlation noted when METS-VF surpassed 4.94 (OR = 34.87, 95% CI: 19.85–64.24). Subgroup analyses by age, ethnicity, and gender indicated that for NAFLD, the positive association with METS-VF was more pronounced in Mexican American female adolescents aged 16 to 19 years, whereas for liver fibrosis, the positive association was stronger in Mexican American female adolescents aged 12 to 16 years. METS-VF was positively associated with the prevalence of NAFLD and hepatic fibrosis in American adolescents. Adolescents with a METS-VF exceeding 5.75 should exercise caution, as higher METS-VF levels may elevate the risk of developing NAFLD and hepatic fibrosis. Additionally, Mexican American female adolescents should be particularly vigilant, as increased METS-VF may heighten the risk of NAFLD and liver fibrosis in this demographic group.

## 1. Introduction

The incidence of nonalcoholic fatty liver disease (NAFLD) has steadily increased in recent years due to changes in diet, lifestyle, and health status, resulting in a significant socio-economic burden (PNAFLD affects approximately 25% of the global population and is the most common chronic liver disease worldwide.^[1]^ It is also the leading cause of chronic liver disease in adults and children in developed countries.^[2]^ Studies indicate that 9.6% of the U.S. population aged 2 to 19 years has NAFLD, with the prevalence rising to nearly 40% among the obese population.^[3]^ NAFLD can progress from simple steatosis to hepatic fibrosis, and may further advance to cirrhosis, increasing the risk of hepatocellular carcinoma.^[4,5]^ A 20-year long-term follow-up study of children with NAFLD revealed that their long-term survival was significantly shorter compared to the expected survival of the general population of the same age and sex. Children with NAFLD had a 13.8-fold higher risk of death or requiring a liver transplant than the general population of the same age and sex.^[6]^ Therefore, given the increasing prevalence of obesity among children and adolescents, it is crucial to identify young patients at risk for advanced fibrosis who may progress to cirrhosis and liver failure.

Early detection and evaluation of NAFLD and liver fibrosis are critical for tracking disease progression and selecting appropriate treatments.^[7]^ Although a pathologic biopsy remains the gold standard for assessing the severity of hepatic steatosis and liver fibrosis, it is invasive, expensive, and carries risks. Therefore, noninvasive methods to identify the severity of NAFLD and liver fibrosis would be highly beneficial. Vibration-controlled transient elastography (VCTE) is the most widely used noninvasive method, which measures liver stiffness (LSM) using a FibroScanⓇ (FS) device to detect fibrosis.^[8]^ Recent observational studies have demonstrated the high accuracy of VCTE in estimating the grade of hepatic steatosis and the stage of liver fibrosis.^[9]^

The risk factors for NAFLD are complex. While obesity is commonly associated with NAFLD, a significant proportion of patients are not obese, presenting a challenge for screening.^[10]^ Recent research suggests that visceral fat more accurately reflects an unfavorable metabolic profile, often linked to abdominal obesity.^[11]^ Several studies have consistently found that higher body mass index (BMI) or larger waist circumference is associated with the presence and severity of liver fibrosis.^[6]^ This implies that abdominal obesity may be more strongly correlated with liver fibrosis. The metabolic evaluation of visceral fat score (METS-VF) is a new index of visceral obesity, developed from a nonlinear fit of the insulin resistance component (METS-IR), waist-to-height ratio (WHtR), age, and sex, using dual X-ray absorptiometry (DXA) as a reference. METS-VF has been validated by magnetic resonance imaging (MRI) and bioelectrical impedance analysis (BIA) for measuring visceral adipose tissue mass in external populations, showing superiority over several other visceral fat surrogates.^[12]^ However, the relationship between METS-VF and liver metrics remains unclear. In this study, we utilized data from the National Health and Nutrition Examination Survey (NHANES) to investigate the association between the METS-VF and hepatic steatosis and liver fibrosis in a population of US adolescents.

## 2. Materials and methods

### 2.1. Data sources

The baseline clinical data evaluated in this study were obtained from the NHANES database covering the years 2017 through 2020. Conducted biennially by the Centers for Disease Control and Prevention (CDC), NHANES is one of the largest cross-sectional surveys in the nation, including approximately 10,000 cases from across the United States. The NHANES study protocol was reviewed and approved by the National Center for Health Statistics (NCHS) Institutional Review Board, and participant consent forms were signed during the survey. Since the NHANES database is publicly available, this study was exempt from additional ethical review.

### 2.2. Participants

**Figure 1.**
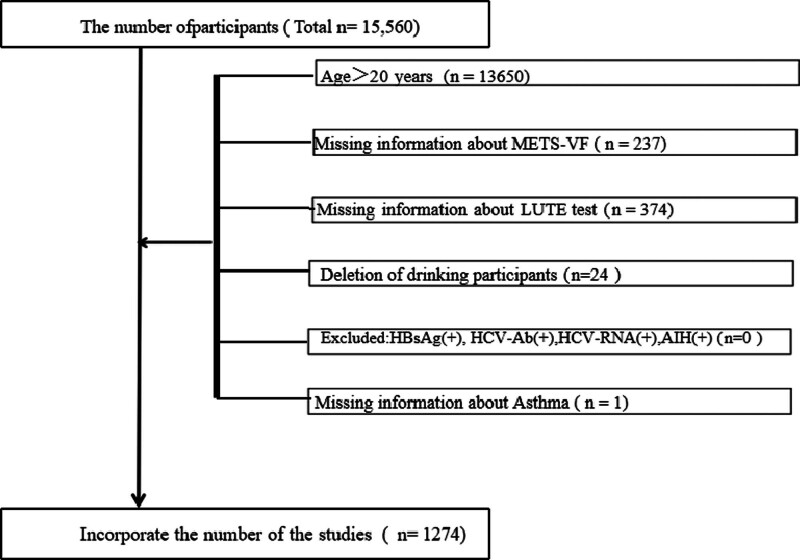
The participants selecting flow chart.

A total of 15,560 participants were initially enrolled in the NHANES database. For this study, we excluded subjects who were older than 20 years (n = 13,650), those without METS-VF information (n = 237), and those who had not completed the LUTE test (n = 374). We also excluded subjects with a history of alcohol consumption (n = 24). Furthermore, participants with a history of viral hepatitis were excluded, including those positive for hepatitis B surface antigen (HBsAg) (n = 0), hepatitis C antibody (HCV-Ab) (n = 0), and hepatitis C virus RNA (HCV-RNA) (n = 0). Subjects with autoimmune hepatitis (AIH) were excluded as well (n = 0). Finally, participants lacking a history of asthma were removed (n = 1). Ultimately, the remaining 1274 participants were included in the study. The specific flow chart is shown in Figure 1.

### 2.3. Evaluation of NAFLD and liver fibrosis

Controlled attenuation parameter (CAP) and liver stiffness measurement (LSM) were used to measure the outcome variables of hepatic steatosis and liver fibrosis, respectively. NHANES staff evaluated participants using VCTE with a FibroScan-equipped Model 502 V2 Touch. A CAP value (also known as CAP) of ≥ 274 dB/m is considered indicative of steatosis, based on a recent landmark study.^[13]^ A median LSM of ≥8.0 kPa is considered indicative of the presence of liver fibrosis (≥F2).^[14]^

### 2.4. Covariate assessment

The METS-VF index can be calculated using the following formula:


$$\begin{array}{l} \;\;\;\begin{array}{l} METS\text{-}VF=4.466+0.011\left[\left(Ln\left(METS\text{-}IR\right)\right)3\right]+3.329\left[\left(Ln\left(WHtR\right)\right)3\right]\;+0.319\left(sex\right)+0.594\left(Ln\left(age\right)\right),\; \end{array} \end{array}$$

where


$$\begin{array}{l} METS\text{-}IR=Ln\left(\left(2\times GLU\right)+TG\right)\times BMI)/\left(Ln\left(HDL\text{-}C\right)\right). \end{array}$$

$$\begin{array}{l} WHtR=WC\left(cm\right)/HT\left(cm\right). \end{array}$$

Age is the participant’s age in years.

Sex is a binary variable, usually coded as 0 for female and 1 for male.

Triglyceride and fasting glucose concentrations were determined enzymatically using an automated biochemical analyzer. Specifically, serum triglyceride concentrations were measured using chemistry analyzers Roche Cobas 6000 and Modular P. These analyzers provide accurate and precise measurements of biochemical parameters. Physical measurements, including height, weight, and waist circumference, were collected by trained health technicians at a mobile examination center. These measurements were obtained using standardized protocols to ensure consistency and reliability across participants.

### 2.5. Covariate

Multivariable-adjusted models were employed to account for potential covariates that could confound the association of the METS-VF index with NAFLD and liver fibrosis. These covariates included demographic factors such as race and household income to poverty ratio, which were obtained through questionnaire information provided by the participants.

Dietary information, including energy intake and sugar intake, was also considered. These data were collected through questionnaires and calculated as the mean of the sum of the values of the ingested substances answered on the first and second day.

Additionally, disease information, primarily related to asthma, was included as a covariate. This information was also obtained through questionnaires.

Laboratory covariates included total cholesterol (mg/dL), C-reactive protein (CRP, mg/L), and creatinine (mg/dL). These biomarkers were measured using standardized laboratory procedures to ensure accuracy and reliability of the data.

Handling of missing values: In this manuscript, there are many missing values for ratio of family income to poverty, total calories, and total sugars. In order to eliminate the selection bias caused by deleting the data,^[15,16]^ we converted these 3 data into categorical variables, and set the missing values into the “unclear” group.

### 2.6. Statistical methods

Data collation and statistical analysis were conducted using R (version 4.1.2) and Empower Stats (version 4.0). Field operations for the NHANES program were suspended in March 2020 due to the 2019 COVID-19 pandemic. Consequently, data collected from 2019 to March 2020 were merged with data from the NHANES 2017 to 2018 cycle to form a nationally representative sample. To address the impact of the pandemic, special weighting was applied by the NHANES workgroup to the pre-pandemic data files from March 2017 to 2020. These data files were weighted in accordance with NHANES guidelines, with particular emphasis on using NHANES check sample weights in the analysis of LUTE data. Therefore, special check sample weights for the 2017 to 2020 cycle (variable name: WTMECPRP) were utilized in this study. The complex multistage stratified sampling technique employed by NHANES was interpreted by utilizing the weights provided in the dataset, utilizing the survey design R package within the R language.

Multiple linear regression analysis was employed to examine the relationship between independent and dependent variables. In this study, given that there were only 54 cases of liver fibrosis, we ensured the logistic regression requirements (number of positive cases/inclusion variable ≥ 10)^[17,18]^ were met by screening the inclusion variables based on 2 criteria^[19,20]^: The inclusion or exclusion of a covariate in the basic or full model resulted in a change of more than 10% in the regression coefficient of the primary predictor (X). The covariate met the first criterion and had a regression coefficient for the outcome variable (Y) with a *P*-value of <.1. Three models were constructed, each adjusting for different covariates:

Model 1: No adjustment for covariates.

Model 2: Adjustment for race.

In NAFLD analyses, Model 3 was adjusted for Model 2 + CRP, creatinine, cholesterol, BMI, asthma, ratio of family income to poverty, total sugars, and total calories; in the liver fibrosis’s analysis, Model 3 was adjusted for Model 2 + cholesterol.

Subgroup analyses were then conducted to identify more sensitive cohorts, assessing the stability of correlations between independent and dependent variables across cohorts and identifying sensitive populations. Smoothed curve-fitting analysis was employed to assess whether a nonlinear relationship existed between independent and dependent variables, utilizing a threshold effects model. For the threshold effect analysis, a log-likelihood ratio (LLR) of <0.05 was used as a criterion for detecting a nonlinear relationship.

## 3. Results

### 3.1. Baseline characteristics

Ultimately, a total of 1274 participants were included in this study. Participants were categorized into 2 groups: group 1 (<4.95) and group 2 (>4.95), based on the median METS-VF value of 4.95. A comparison between these groups revealed that participants with METS-VF > 4.95 exhibited a higher incidence of NAFLD (*P* < .05). However, while there was a tendency for the incidence of liver fibrosis to increase in the METS-VF group, this trend did not reach statistical significance due to limitations in sample size. The results of participant characteristics are summarized in Table 1. In addition to this, we additionally grouped the variables in the manuscript by the presence of NAFLD and liver fibrosis in order to demonstrate clearly the specific information of each variable, and the results are displayed in Tables 2 and 3, respectively.

**Table 1:** Baselines characteristics of participants, weighted.

Characteristics	Group 1	Group 2	*P*-value
Sample size	637	638	
Age (yr)	14.99 (14.78,15.21)	15.89 (15.69,16.09)	<.0001
BMI (kg/m^2^)	20.13 (19.96,20.30)	28.96 (28.47,29.46)	<.0001
Serum creatinine (mg/dL)	0.71 (0.69,0.72)	0.72 (0.71,0.74)	.1142
Serum cholesterol (mg/dL)	151.46 (148.07,154.85)	157.27 (154.04,160.50)	.0102
CRP (mg/L)	1.19 (0.87,1.51)	3.00 (2.52,3.49)	<.0001
Gender (%)			
Male	52.01 (45.15,58.80)	53.20 (47.90,58.43)	.7363
Female	47.99 (41.20,54.85)	46.80 (41.57,52.10)	
Race (%)			
Mexican American	11.48 (8.01,16.19)	22.31 (16.02,30.19)	.0001
White	64.16 (57.18,70.59)	57.37 (47.73,66.47)	
Black	13.33 (9.83,17.83)	10.39 (6.65,15.85)	
Other race	11.02 (8.16,14.72)	9.93 (7.39,13.24)	
Asthma (%)			
Yes	20.21 (15.83,25.44)	20.83 (17.12,25.09)	.688
No	79.79 (74.56,84.17)	79.06 (74.82,82.75)	
Stratified by LSM (kPa) (%)			
<8.0	97.36 (95.77,98.36)	96.06 (91.84,98.15)	.3224
≥8.0	2.64 (1.64,4.23)	3.94 (1.85,8.16)	
Stratified by CAP (dB/m) (%)			
<274	97.87 (95.83,98.92)	71.19 (65.52,76.26)	<.0001
≥274	2.13 (1.08,4.17)	28.81 (23.74,34.48)	
PIR (%)			
<1.3	19.75 (16.19,23.86)	33.50 (27.13,40.54)	<.0001
≥1.3 < 3.5	35.19 (29.72,41.07)	31.89 (25.98,38.45)	
≥3.5	37.45 (32.11,43.12)	24.81 (20.48,29.70)	
Unclear	7.61 (5.13,11.15)	9.80 (6.98,13.61)	
Total sugar (%)			
Lower	34.39 (29.84,39.25)	42.64 (37.37,48.08)	.0372
Higher	46.46 (40.50,52.53)	40.42 (35.90,45.10)	
Unclear	19.14 (15.06,24.03)	16.95 (13.57,20.96)	
Total (Kcal)			
Lower	37.47 (31.85,43.46)	43.82 (38.66,49.11)	.129
Higher	43.38 (38.90,47.98)	39.24 (34.82,43.84)	
Unclear	19.14 (15.06,24.03)	16.95 (13.57,20.96)	

Note: Data of continuous variables are shown as survey-weighted mean (95% CI), *P*-value was calculated by survey-weighted linear regression. Data of categorical variables are shown as survey-weighted percentage (95% CI), *P*-value was calculated by survey-weighted Chi-square test..

Abbreviations: BMI = body mass index, CAP = controlled attenuation parameter, CRP = C-reactive protein, LSM = liver stiffness measurement.

**Table 2:** Baseline data comparisons were classified according to whether liver fibrosis was present or not.

Liver fibrosis	No	Yes	*P*-value
N	1220	54	
Age (yr)	15.50 ± 2.23	16.07 ± 2.17	.061
BMI (kg/m^2^)	25.11 ± 6.09	31.51 ± 12.54	.005
Serum creatinine (mg/dL)	0.72 ± 0.17	0.73 ± 0.16	.458
Serum cholesterol (mg/dL)	155.47 ± 30.11	148.28 ± 26.57	.076
METS-VF	5.01 ± 0.70	5.26 ± 0.98	.008
CRP (mg/L)	2.10 ± 5.15	3.74 ± 4.63	.007
Gender (%)			
Male	642 (52.62%)	35 (64.81%)	.079
Female	578 (47.38%)	19 (35.19%)	
Race (%)			
Mexican American	206 (16.89%)	8 (14.81%)	.003
White	532 (43.61%)	14 (25.93%)	
Black	252 (20.66%)	22 (40.74%)	
Other race	230 (18.85%)	10 (18.52%)	
NAFLD (%)			
No	1025 (84.02%)	28 (51.85%)	<.001
Yes	195 (15.98%)	26 (48.15%)	
PIR (%)			
<1.3	429 (35.16%)	23 (42.59%)	.041
≥1.3 < 3.5	380 (31.15%)	18 (33.33%)	
≥3.5	281 (23.03%)	4 (7.41%)	
Unclear	130 (10.66%)	9 (16.67%)	
Total sugar (%)			
Lower	491 (40.25%)	29 (53.70%)	.061
Higher	509 (41.72%)	14 (25.93%)	
Unclear	220 (18.03%)	11 (20.37%)	
Total (Kcal) (%)			
Lower	490 (40.16%)	29 (53.70%)	.059
Higher	510 (41.80%)	14 (25.93%)	
Unclear	220 (18.03%)	11 (20.37%)	
Asthma (%)			
Yes	249 (20.41%)	12 (22.22%)	.747
No	971 (79.59%)	42 (77.78%)	

Abbreviations: BMI = body mass index, CRP = C-reactive protein, METS-VF = metabolic evaluation of visceral fat score, NAFLD = nonalcoholic fatty liver disease.

**Table 3:** Baseline data comparisons were classified according to whether NAFLD was present or not.

NAFLD	No	Yes	*P*-value
N	1053	221	
Age (yr)	15.45 ± 2.25	15.89 ± 2.06	.009
BMI (kg/m^2^)	23.78 ± 5.10	33.00 ± 7.61	<.001
Serum creatinine (mg/dL)	0.72 ± 0.17	0.73 ± 0.16	.852
Serum cholesterol (mg/dL)	153.78 ± 29.87	161.76 ± 29.73	<.001
METS-VF	4.86 ± 0.64	5.78 ± 0.50	<.001
CRP (mg/L)	1.85 ± 5.17	3.71 ± 4.73	<.001
Gender (%)			
Male	550 (52.23%)	127 (57.47%)	.156
Female	503 (47.77%)	94 (42.53%)	
Race (%)			
Mexican American	158 (15.00%)	56 (25.34%)	.001
White	469 (44.54%)	77 (34.84%)	
Black	225 (21.37%)	49 (22.17%)	
Other race	201 (19.09%)	39 (17.65%)	
Liver fibrosis (%)			
No	1025 (97.34%)	195 (88.24%)	<.001
Yes	28 (2.66%)	26 (11.76%)	
PIR (%)			
<1.3	352 (33.43%)	100 (45.25%)	<.001
≥1.3 < 3.5	330 (31.34%)	68 (30.77%)	
≥3.5	258 (24.50%)	27 (12.22%)	
Unclear	113 (10.73%)	26 (11.76%)	
Total sugar (%)			
Lower	415 (39.41%)	105 (47.51%)	.083
Higher	442 (41.98%)	81 (36.65%)	
Unclear	196 (18.61%)	35 (15.84%)	
Total (Kcal) (%)			
Lower	415 (39.41%)	104 (47.06%)	.108
Higher	442 (41.98%)	82 (37.10%)	
Unclear	196 (18.61%)	35 (15.84%)	
Asthma (%)			
Yes	222 (21.08%)	39 (17.65%)	.25
No	831 (78.92%)	182 (82.35%)	

Abbreviations: BMI = body mass index, CRP = C-reactive protein, METS-VF = metabolic evaluation of visceral fat score, NAFLD = nonalcoholic fatty liver disease.

### 3.2. Higher prevalence of NAFLD and liver fibrosis associated with higher METS-VF index

According to the results presented in Table 4, METS-VF exhibited a positive association with both NAFLD and liver fibrosis across all models. Specifically, the OR for the positive effect between METS-VF and NAFLD was 15.74 (95% CI: 10.44–23.72) in all participants. Similarly, the positive effect between METS-VF and liver fibrosis was observed with an OR of 1.85 (95% CI: 1.27–2.70).

**Table 4:** Logistic regression analysis between METS-VF index with NAFLD and liver fibrosis prevalence.

Characteristic	Model 1 OR (95% CI)	Model 2 OR (95% CI)	Model 3 OR (95% CI)
NAFLD			
METS-VF	16.39 (11.17, 24.04)	16.52 (11.17, 24.45)	15.74 (10.44, 23.74)
Categories			
Lower (2.66–4.53)	1	1	1
Middle (4.53–5.37)	3.10 (1.30, 7.36)	3.07 (1.29, 7.31)	3.04 (1.27, 7.28)
Higher (5.37–6.51)	47.15 (21.85, 101.73)	46.24 (21.37, 100.03)	41.21 (18.88, 89.95)
*P*-value for trend	<.01	<.01	<.01
Liver fibrosis			
METS-VF	1.86 (1.28, 2.71)	1.91 (1.32, 2.76)	1.85 (1.27, 2.70)
Categories			
Lower (2.66–4.53)	1	1	1
Middle (4.53–5.37)	0.59 (0.26, 1.37)	0.71 (0.30, 1.66)	0.78 (0.33, 1.86)
Higher (5.37–6.51)	2.07 (1.10, 3.90)	2.32 (1.21, 4.43)	2.25 (1.14, 4.45)
*P*-value for trend	<.01	<.01	<.01

Note: Model 1 was adjusted for no covariates; Model 2 was adjusted for race; In NAFLD’s analyses, model 3 was adjusted for model 2 + CRP, creatinine, cholesterol, BMI, asthma, PIR, total sugars, and total calories; in the Liver fibrosis analysis, Model 3 was adjusted for Model 2 + cholesterol.

Abbreviations: METS-VF = metabolic evaluation of visceral fat score, NAFLD = nonalcoholic fatty liver disease, OR = odds ratio.

Furthermore, when METS-VF was grouped according to tertiles, the correlation with NAFLD and liver fibrosis remained significant. Moreover, the correlation of METS-VF with both NAFLD and liver fibrosis demonstrated a clear trend of increasing strength with higher METS-VF values (*P* < .01 for trend). Specifically, in the third quartile of METS-VF, there was a particularly strong positive effect between METS-VF and NAFLD (OR = 41.21, 95% CI: 18.88–89.95) as well as with liver fibrosis (OR = 2.25, 95% CI: 1.14–4.45).

### 3.3. Smooth curve fitting and threshold effect analysis

**Figure 2.**
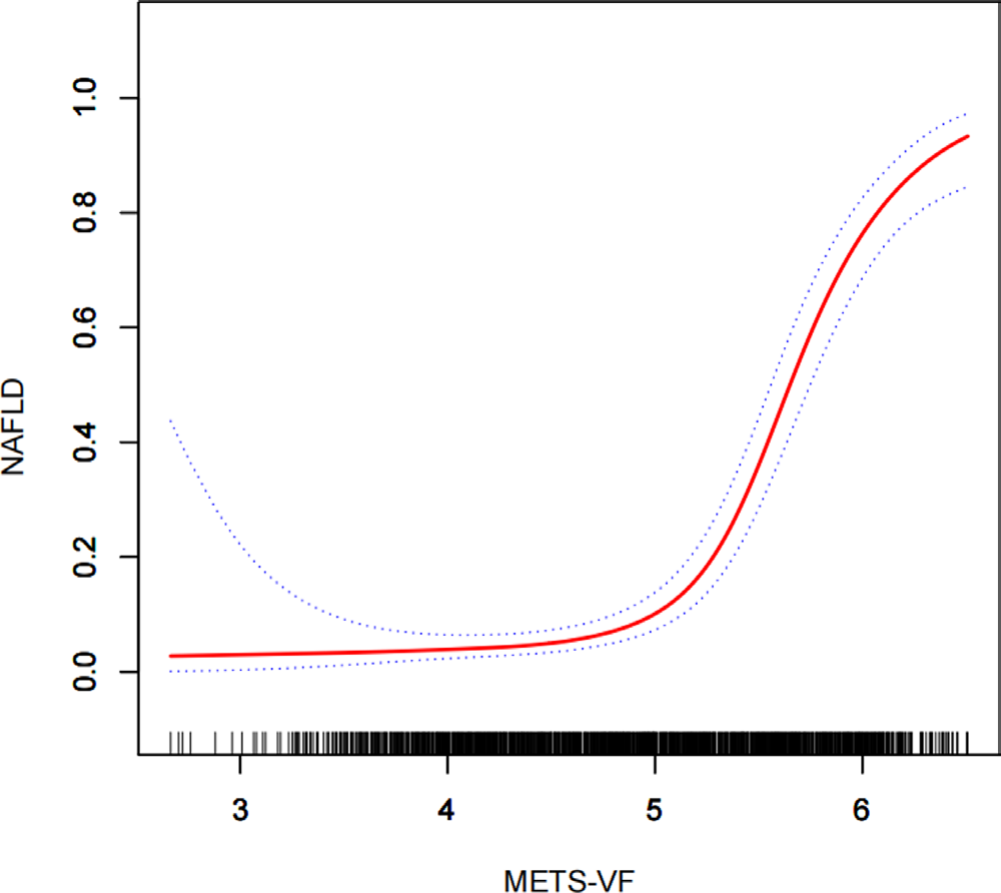
Density dose-response relationship between METS-VF index with NAFLD prevalence. The area between the upper and lower dashed lines is represented as 95% CI. Each point shows the magnitude of the METS-VF index and is connected to form a continuous line. Adjusted for all covariates except effect modifier. METS-VF = metabolic evaluation of visceral fat score, NAFLD = nonalcoholic fatty liver disease

**Figure 3.**
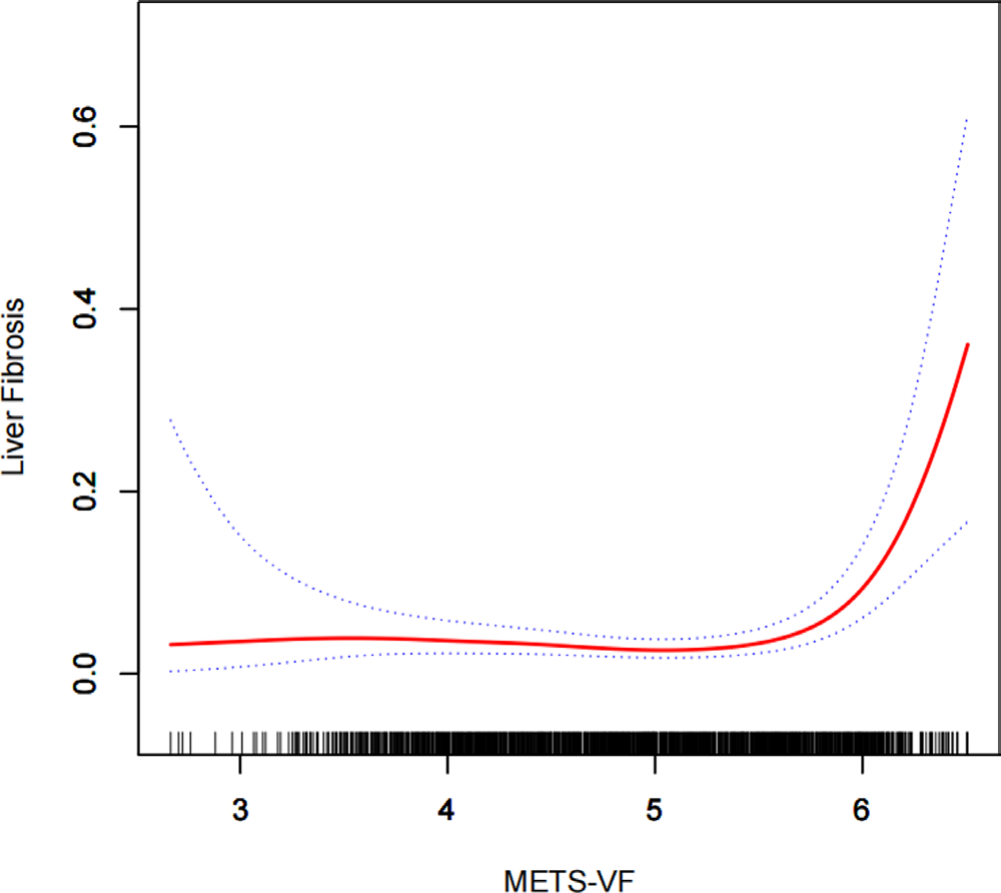
Density dose-response relationship between METS-VF index with liver fibrosis prevalence. The area between the upper and lower dashed lines is represented as 95% CI. Each point shows the magnitude of the METS-VF index and is connected to form a continuous line. Adjusted for gender, race, and cholesterol. METS-VF = metabolic evaluation of visceral fat score.

In our exploration using smoothed curve-fitting analysis, we aimed to determine whether the positive correlation of METS-VF with NAFLD and liver fibrosis followed a linear trend or exhibited nonlinearity, which we verified through a threshold effect. As depicted in Figure 2, we observed that there was no perfect linear association between METS-VF and either of the 2 dependent variables. The results of the threshold effect model revealed a nonlinear correlation between METS-VF and NAFLD. Specifically, the positive correlation between METS-VF and NAFLD became significantly more pronounced at METS-VF values >5.75, with an effect value of OR = 104.42 (95% CI: 17.40–626.58) (Table 5). Similarly, we identified a nonlinear correlation between METS-VF and liver fibrosis (Fig. 3). The positive correlation between METS-VF and liver fibrosis was notably stronger at METS-VF values >4.94, with an effect value of OR = 34.87 (95% CI: 19.85–64.24) (Table 5).

**Table 5:** Threshold effect analysis for association of METS-VF with NAFLD and liver fibrosis.

Outcomes	NAFLD	Liver fibrosis
Model 1, β (95% CI)		
Linear effect model	15.74 (10.44, 23.74)	1.83 (1.27, 2.65)
Model 2, β (95% CI)		
Inflection point (K)	4.94	5.75
<K	1.64 (0.69, 3.86)	0.86 (0.54, 1.37)
>K	34.87 (19.85, 61.24)	104.42 (17.40, 626.58)
LLR	<.001	<.001

Abbreviations: LLR = log-likelihood ratio, METS-VF = metabolic evaluation of visceral fat score, NAFLD = nonalcoholic fatty liver disease.

### 3.4. Subgroup analysis

In subsequent subgroup analyses, we examined the influence of gender, age, and race on the correlation between METS-VF and NAFLD as well as liver fibrosis. First, focusing on gender, we observed that the positive correlation of METS-VF with both NAFLD and liver fibrosis remained consistent across all genders. However, the correlation appeared to be more pronounced in female participants compared to male participants. Second, when dividing participants into 2 age groups, we found that the positive correlation between METS-VF and NAFLD remained stable within the 16 to 19 age group. Similarly, the positive correlation between METS-VF and liver fibrosis remained stable across all age groups. Finally, regarding race, we discovered that the positive correlation between METS-VF and NAFLD with hepatic fibrosis was notably more pronounced in participants of Mexican origin. This suggests that the relationship between METS-VF and the studied conditions may vary across different racial groups. The results of subgroup analyses of the association of METS-VF with NAFLD and liver fibrosis are shown in Tables 6 and 7, respectively.

**Table 6:** Subgroup regression analysis between METS-VF index with NAFLD prevalence.

Characteristic	Model 1 OR (95% CI)	Model 2 OR (95% CI)	Model 3 OR (95% CI)
Stratified by gender			
Male	14.53 (8.94, 23.63)	14.20 (8.71, 23.15)	15.34 (9.06, 25.95)
Female	19.77 (10.55, 37.05)	20.70 (10.88, 39.39)	19.80 (9.87, 39.72)
Stratified by race			
Mexican American	13.97 (6.14, 31.76)	12.84 (5.61, 29.38)	10.82 (4.48, 26.11)
White people	21.53 (10.82, 42.84)	22.78 (11.29, 45.96)	27.58 (12.00, 63.39)
Black people	11.18 (5.57, 22.43)	11.16 (5.59, 22.28)	10.30 (4.77, 22.26)
Other race	21.61 (8.32, 56.14)	21.76 (8.20, 57.72)	29.58 (9.35, 93.61)
Stratified by age (yr)			
12 to 15	21.99 (11.81, 40.93)	24.42 (12.61, 47.27)	20.78 (10.53, 41.00)
16 to 19	14.09 (8.55, 23.22)	14.00 (8.41, 23.31)	14.59 (8.31, 25.64)

Note: Model 1 was adjusted for no covariates; Model 2 was adjusted for race; In NAFLD’s analyses, model 3 was adjusted for model 2 + CRP, creatinine, cholesterol, BMI, asthma, PIR, total sugars and total calories.

Abbreviations: METS-VF = metabolic evaluation of visceral fat score, NAFLD = nonalcoholic fatty liver disease, OR = odds ratio.

**Table 7:** Subgroup regression analysis between METS-VF index with liver fibrosis prevalence.

Characteristic	Model 1 OR (95% CI)	Model 2 OR (95% CI)	Model 3 OR (95% CI)
Stratified by gender			
Male	1.38 (0.91, 2.10)	1.46 (0.97, 2.22)	1.60 (1.02, 2.49)
Female	4.54 (1.90, 10.87)	4.15 (1.76, 9.78)	2.98 (1.33, 6.68)
Stratified by race			
Mexican American	11.28 (1.94, 65.62)	9.00 (1.50, 54.07)	8.18 (1.26, 53.16)
White people	2.24 (1.02, 4.92)	2.23 (1.01, 4.90)	2.27 (0.96, 5.35)
Black people	1.27 (0.78, 2.07)	1.31 (0.80, 2.14)	1.26 (0.76, 2.08)
Other race	2.68 (1.03, 7.00)	2.40 (0.94, 6.18)	3.60 (1.19, 10.85)
Stratified by age (yr)			
12 to 15	1.06 (0.62, 1.81)	1.15 (0.68, 1.94)	1.02 (0.60, 1.74)
16 to 19	2.93 (1.67, 5.16)	2.94 (1.69, 5.10)	2.82 (1.60, 4.98)

Note: Model 1 was adjusted for no covariates; Model 2 was adjusted for race; In the Liver fibrosis analysis, Model 3 was adjusted for Model 2 + cholesterol.

Abbreviations: METS-VF = metabolic evaluation of visceral fat score, NAFLD = nonalcoholic fatty liver disease, OR = odds ratio.

## 4. Discussion

This pioneering cross-sectional study represents the first examination of the association between METS-VF and the prevalence of NAFLD and hepatic fibrosis within a representative sample of US adolescents. Our findings underscore a clear and positive correlation between METS-VF and the prevalence of both steatosis and hepatic fibrosis among adolescents in the United States. Moreover, we observed that this positive association becomes more pronounced with higher METS-VF values, suggesting a dose-response relationship. In this study, we also determined that there is a threshold effect of METS-VF on NAFLD and liver fibrosis, and that when the METS-VF value is >4.94, the probability of NAFLD will be much higher, and when the METS-VF value reaches 5.75, there will be a possibility of liver fibrosis complication. These results contribute to our understanding of metabolic health among adolescents and emphasize the importance of early identification and intervention strategies to address the growing prevalence of NAFLD and hepatic fibrosis in this population. Further research is warranted to elucidate the underlying mechanisms driving this association and to develop targeted interventions aimed at reducing the burden of metabolic liver diseases in adolescents.

In a recent study focusing on an adolescent discovery population, it was observed that moderate to severe fibrosis correlated with higher levels of BMI and moderate to severe steatosis.^[21]^ However, the conventional use of BMI as an indicator for assessing obesity has been called into question. Major limitations of BMI include its inability to differentiate between fat mass and lean mass, as well as its failure to account for localized fat distribution patterns.^[22]^ To provide a more accurate representation of obesity, a novel obesity index named METS-VF was proposed. Several studies have demonstrated the relationship between abdominal obesity and the presence of hepatic steatosis or fibrosis. A longitudinal study conducted in Catalonia identified abdominal obesity and dysglycemia as primary metabolic risk factors associated with the progression to moderate to advanced hepatic fibrosis in both the general population and individuals with NAFLD.^[23]^ Additionally, Chinese researchers found a positive correlation between the weight-adjusted waist index and the prevalence of NAFLD and hepatic fibrosis in US adults.^[24]^ However, recent data suggest that adult scoring systems may not accurately predict advanced fibrosis in children,^[25–27]^ highlighting the necessity for evaluating noninvasive methods for diagnosing liver fibrosis in pediatric populations. The significant association observed between METS-VF and NAFLD as well as hepatic fibrosis in the adolescent population underscores the potential of METS-VF as a predictive tool for these conditions in adolescents. Nonetheless, the stability of this finding warrants confirmation through multicenter, large-sample prospective cohort studies.

Furthermore, our study confirmed a positive correlation between METS-VF and the dependent variables, NAFLD or liver fibrosis, within sensitive populations. Gender was the first validated characteristic. Previous epidemiological studies have suggested that increased adiposity may play a more significant role in the development of hepatic fibrosis in women with abdominal obesity patterns.^[28]^ A Taiwanese study also identified significant correlations between metabolic syndrome and obesity-related indices and NAFLD, with indices such as metabolic syndrome, waist-to-hip ratio, lipid accumulation products, and triglyceride-glucose index correlating more significantly with NAFLD in women compared to men.^[29]^ This discrepancy may be attributed to the regulatory role of estrogen in adipose tissue development and deposition in females, potentially promoting the accumulation of subcutaneous adipose tissue and leading to a greater increase in subcutaneous and total body fat in females compared to males.^[30]^ Our study revealed that METS-VF had a stronger effect on the prevalence of NAFLD in adolescents aged 12 to 16 years compared to those aged 16 to 19 years. This finding holds significant clinical implications as it suggests that NAFLD onset may occur at an earlier age, potentially leading to earlier hepatic fibrosis development. This underscores the importance of clinical vigilance regarding the adverse effects of METS-VF on adolescents. Finally, our study also identified racial disparities in NAFLD and liver fibrosis within the US population, with METS-VF exerting the strongest effect on adolescents of Mexican descent. These findings align with research conducted by Nobili et al,^[31]^ who mapped the prevalence of NAFLD in children and reported the highest prevalence in Central America and the Middle East. Specifically, in Mexico, the prevalence of NAFLD in children aged 8 to 11 years was reported to be 42.5%, and in children under 20 years of age, it was 16.9%, as measured by alanine aminotransferase assays. Additionally, a study from the United States differentiated between Hispanic American populations and found that Mexican Hispanics exhibited a higher prevalence of NAFLD compared to Hispanics of Dominican and Puerto Rican descent. Even after controlling for traditional risk factors such as diabetes mellitus and metabolic syndrome, Mexican Hispanics still had a higher likelihood of NAFLD, with genetic differences, particularly the PNPLA3 gene, explaining up to 72% of the racial difference in NAFLD prevalence.^[32]^

While there have been several reports exploring the mechanisms underlying obesity and its association with NAFLD and liver fibrosis, further research is still required to fully understand these complex interactions. Obesity is known to induce oxidative stress and chronic low-grade inflammation within the body.^[33,34]^ Reactive oxygen species play a crucial role in this process, initiating a cascade of oxidative events that contribute to liver injury and the progression of NAFLD.^[35]^ Reactive oxygen species stimulate lipid peroxidation, particularly of polyunsaturated fatty acids, leading to the formation of highly reactive aldehyde products such as malondialdehyde and 4-hydroxy-2-nonenal (4-HNE).^[36]^ Additionally, oxidative stress can indirectly or directly promote the upregulation of nuclear factor κ-light chain enhancers of activated B cells (nuclear factor kappa-B) and pro-inflammatory cytokines (tumor necrosis factor alpha, interleukin-6, and interleukin-1), which are implicated in apoptosis and the development of liver fibrosis.^[37]^ Animal experiments, such as those conducted on KK-Ay mice fed a high-fat, high-fructose, and high-cholesterol diet supplemented with bile acids, have provided insights into the pathogenesis of NAFLD. These mice developed severe obesity, insulin resistance, and dyslipidemia, exhibiting significant steatohepatitis within 4 weeks and substantial fibrosis within 12 weeks.^[38]^ Furthermore, obesity has been associated with reduced levels of lipocalin, an observation observed in patients with NAFLD and correlated with advanced fibrosis.^[39,40]^ Hypolipocalinemia has also been observed in NAFLD mouse models.^[38,41]^

Our study holds the distinction of being the inaugural cross-sectional investigation to explore the correlation between visceral fat distribution, as represented by METS-VF, and the prevalence of both NAFLD and hepatic fibrosis. Importantly, the study boasts an ample and representative sample size, enhancing the robustness and generalizability of our findings. The subgroup analyses conducted provide valuable insights into the nuanced characteristics of different age groups, genders, and ethnicities concerning this correlation, thereby offering potential guidance for tailored clinical recommendations across diverse populations. Nevertheless, it is crucial to acknowledge certain limitations inherent in our study. First, as a cross-sectional study, we are unable to establish a causal relationship between METS-VF and NAFLD or liver fibrosis. Further research is warranted to elucidate whether such a relationship exists and, if so, whether it is unidirectional or bidirectional. Second, while shear wave elastography was utilized to assess NAFLD and liver fibrosis, the absence of liver biopsy, which remains the gold standard for NAFLD diagnosis, represents a limitation. Third, numerous factors may influence METS-VF in conjunction with NAFLD and liver fibrosis. Despite our efforts to incorporate relevant covariates into our model, it is conceivable that other potential covariates could impact our findings. Notwithstanding these limitations, we assert that our study underscores a positive association between heightened METS-VF and the prevalence of both NAFLD and liver fibrosis.

## 5. Summary

The positive association observed between METS-VF and the prevalence of NAFLD and hepatic fibrosis among American adolescents underscores the importance of monitoring METS-VF levels in this population. Adolescents with a METS-VF surpassing 5.75 should exercise caution, as elevated METS-VF levels may increase the likelihood of developing NAFLD and hepatic fibrosis. Furthermore, special attention should be paid to Mexican American female adolescents, as heightened METS-VF levels may further elevate their risk of NAFLD and liver fibrosis. Vigilance in monitoring and managing METS-VF levels in this demographic group could aid in mitigating the risk of these hepatic complications.

## Author contributions

**Conceptualization:** Xiaomei Xu.

**Data curation:** Xiaomei Xu.

**Formal analysis:** Xiaomei Xu.

**Investigation:** Xiaomei Xu, Junping Yang.

**Methodology:** Junping Yang, Yang Li.

**Resources:** BIquan Chen.

**Software:** Junping Yang, Xiaoyan Zeng.

**Supervision:** Yang Li.

**Validation:** BIquan Chen.

**Visualization:** Xiaoyan Zeng.

**Writing – original draft:** Yuanyuan Li, BIquan Chen.

**Writing – review & editing:** BIquan Chen.

## References

[1] Huang DQ El-Serag HB Loomba R. Global epidemiology of NAFLD-related HCC: trends, predictions, risk factors and prevention. Nat Rev Gastroenterol Hepatol. 2021;18:223–38. 33349658 10.1038/s41575-020-00381-6 PMC8016738

[2] Chalasani N Younossi Z Lavine JE; American Gastroenterological Association. The diagnosis and management of non-alcoholic fatty liver disease: practice guideline by the American Gastroenterological Association, American Association for the Study of Liver Diseases, and American College of Gastroenterology. Gastroenterology. 2012;142:1592–609. 22656328 10.1053/j.gastro.2012.04.001

[3] Schwimmer JB Deutsch R Kahen T Lavine JE Stanley C Behling C. Prevalence of fatty liver in children and adolescents. Pediatrics. 2006;118:1388–93. 17015527 10.1542/peds.2006-1212

[4] Imajo K Fujita K Yoneda M. Hyperresponsivity to low-dose endotoxin during progression to nonalcoholic steatohepatitis is regulated by leptin-mediated signaling. Cell Metab. 2012;16:44–54. 22768838 10.1016/j.cmet.2012.05.012

[5] Meex RC Hoy AJ Morris A. Fetuin B is a secreted hepatocyte factor linking steatosis to impaired glucose metabolism. Cell Metab. 2015;22:1078–89. 26603189 10.1016/j.cmet.2015.09.023

[6] Feldstein AE Charatcharoenwitthaya P Treeprasertsuk S Benson JT Enders FB Angulo P. The natural history of non-alcoholic fatty liver disease in children: a follow-up study for up to 20 years. Gut. 2009;58:1538–44. 19625277 10.1136/gut.2008.171280 PMC2792743

[7] Friedman SL Neuschwander-Tetri BA Rinella M Sanyal AJ. Mechanisms of NAFLD development and therapeutic strategies. Nat Med. 2018;24:908–22. 29967350 10.1038/s41591-018-0104-9 PMC6553468

[8] Hwang JY Yoon HM Kim JR. Diagnostic performance of transient elastography for liver fibrosis in children: a systematic review and meta-analysis. AJR Am J Roentgenol. 2018;211:W257–66. 30106615 10.2214/AJR.18.19535

[9] Siddiqui MS Vuppalanchi R Van Natta ML; NASH Clinical Research Network. Vibration-controlled transient elastography to assess fibrosis and steatosis in patients with nonalcoholic fatty liver disease. Clin Gastroenterol Hepatol. 2019;17:156–63.e2. 29705261 10.1016/j.cgh.2018.04.043 PMC6203668

[10] Younossi Z Anstee QM Marietti M. Global burden of NAFLD and NASH: trends, predictions, risk factors and prevention. Nat Rev Gastroenterol Hepatol. 2018;15:11–20. 28930295 10.1038/nrgastro.2017.109

[11] Thomas EL Frost G Taylor-Robinson SD Bell JD. Excess body fat in obese and normal-weight subjects. Nutr Res Rev. 2012;25:150–61. 22625426 10.1017/S0954422412000054

[12] Bello-Chavolla OY Antonio-Villa NE Vargas-Vázquez A. Metabolic Score for Visceral Fat (METS-VF), a novel estimator of intra-abdominal fat content and cardio-metabolic health. Clin Nutr. 2020;39:1613–21. 31400997 10.1016/j.clnu.2019.07.012

[13] Eddowes PJ Sasso M Allison M. Accuracy of FibroScan controlled attenuation parameter and liver stiffness measurement in assessing steatosis and fibrosis in patients with nonalcoholic fatty liver disease. Gastroenterology. 2019;156:1717–30. 30689971 10.1053/j.gastro.2019.01.042

[14] Roulot D Czernichow S Le Clésiau H Costes JL Vergnaud AC Beaugrand M. Liver stiffness values in apparently healthy subjects: influence of gender and metabolic syndrome. J Hepatol. 2008;48:606–13. 18222014 10.1016/j.jhep.2007.11.020

[15] Liu Q Han X Chen Y Gao Y Yang W Huang L. Asthma prevalence is increased in patients with high metabolism scores for visceral fat: study reports from the US. Front Endocrinol (Lausanne). 2023;14:1162158. 37260450 10.3389/fendo.2023.1162158 PMC10227585

[16] Yu L Chen Y Xu M. Association of weight-adjusted-waist index with asthma prevalence and the age of first asthma onset in United States adults. Front Endocrinol (Lausanne). 2023;14:1116621. 36896186 10.3389/fendo.2023.1116621 PMC9988541

[17] Stoltzfus JC. Logistic regression: a brief primer. Acad Emerg Med. 2011;18:1099–104. 21996075 10.1111/j.1553-2712.2011.01185.x

[18] Peduzzi P Concato J Kemper E Holford TR Feinstein AR. A simulation study of the number of events per variable in logistic regression analysis. J Clin Epidemiol. 1996;49:1373–9. 8970487 10.1016/s0895-4356(96)00236-3

[19] Jaddoe VW de Jonge LL Hofman A Franco OH Steegers EA Gaillard R. First trimester fetal growth restriction and cardiovascular risk factors in school age children: population based cohort study. BMJ. 2014;348:g14. 24458585 10.1136/bmj.g14 PMC3901421

[20] Bjerregaard LG Pedersen DC Mortensen EL Sørensen TIA Baker JL. Breastfeeding duration in infancy and adult risks of type 2 diabetes in a high-income country. Matern Child Nutr. 2019;15:e12869. 31267694 10.1111/mcn.12869 PMC6860000

[21] Moran-Lev H Cohen S Webb M. Higher BMI predicts liver fibrosis among obese children and adolescents with NAFLD - an interventional pilot study. BMC Pediatr. 2021;21:385. 34479517 10.1186/s12887-021-02839-1 PMC8414665

[22] Dixon AE Peters U. The effect of obesity on lung function. Expert Rev Respir Med. 2018;12:755–67. 30056777 10.1080/17476348.2018.1506331 PMC6311385

[23] Julián MT Ballesta S Pera G. Abdominal obesity and dsyglycemia are risk factors for liver fibrosis progression in NAFLD subjects: a population-based study. Front Endocrinol (Lausanne). 2022;13:1051958. 36714592 10.3389/fendo.2022.1051958 PMC9880071

[24] Hu Q Han K Shen J Sun W Gao L Gao Y. Association of weight-adjusted-waist index with non-alcoholic fatty liver disease and liver fibrosis: a cross-sectional study based on NHANES. Eur J Med Res. 2023;28:263. 37537679 10.1186/s40001-023-01205-4 PMC10399060

[25] Vos MB Abrams SH Barlow SE. NASPGHAN clinical practice guideline for the diagnosis and treatment of nonalcoholic fatty liver disease in children: recommendations from the expert committee on NAFLD (ECON) and the North American Society of Pediatric Gastroenterology, Hepatology and Nutrition (NASPGHAN). J Pediatr Gastroenterol Nutr. 2017;64:319–34. 28107283 10.1097/MPG.0000000000001482 PMC5413933

[26] Roberts EA. Pediatric nonalcoholic fatty liver disease (NAFLD): a “growing” problem. J Hepatol. 2007;46:1133–42. 17445934 10.1016/j.jhep.2007.03.003

[27] Freedman DS Mei Z Srinivasan SR Berenson GS Dietz WH. Cardiovascular risk factors and excess adiposity among overweight children and adolescents: the Bogalusa Heart Study. J Pediatr. 2007;150:12–7.e2. 17188605 10.1016/j.jpeds.2006.08.042

[28] Ciardullo S Oltolini A Cannistraci R Muraca E Perseghin G. Sex-related association of nonalcoholic fatty liver disease and liver fibrosis with body fat distribution in the general US population. Am J Clin Nutr. 2022;115:1528–34. 35244676 10.1093/ajcn/nqac059

[29] Lin IT Lee MY Wang CW Wu DW Chen SC. Gender differences in the relationships among metabolic syndrome and various obesity-related indices with nonalcoholic fatty liver disease in a Taiwanese population. Int J Environ Res Public Health. 2021;18:857. 33498329 10.3390/ijerph18030857 PMC7908550

[30] Cooke PS Naaz A. Role of estrogens in adipocyte development and function. Exp Biol Med (Maywood). 2004;229:1127–35. 15564439 10.1177/153537020422901107

[31] Anderson EL Howe LD Jones HE Higgins JP Lawlor DA Fraser A. The prevalence of non-alcoholic fatty liver disease in children and adolescents: a systematic review and meta-analysis. PLoS One. 2015;10:e0140908. 26512983 10.1371/journal.pone.0140908 PMC4626023

[32] Romeo S Huang-Doran I Baroni MG Kotronen A. Unravelling the pathogenesis of fatty liver disease: patatin-like phospholipase domain-containing 3 protein. Curr Opin Lipidol. 2010;21:247–52. 20480550 10.1097/mol.0b013e328338ca61

[33] Grasemann H Holguin F. Oxidative stress and obesity-related asthma. Paediatr Respir Rev. 2021;37:18–21. 32660723 10.1016/j.prrv.2020.05.004

[34] Li H Ren J Li Y Wu Q Wei J. Oxidative stress: the nexus of obesity and cognitive dysfunction in diabetes. Front Endocrinol (Lausanne). 2023;14:1134025. 37077347 10.3389/fendo.2023.1134025 PMC10107409

[35] Rector RS Thyfault JP Uptergrove GM. Mitochondrial dysfunction precedes insulin resistance and hepatic steatosis and contributes to the natural history of non-alcoholic fatty liver disease in an obese rodent model. J Hepatol. 2010;52:727–36. 20347174 10.1016/j.jhep.2009.11.030 PMC3070177

[36] Delli Bovi AP Marciano F Mandato C Siano MA Savoia M Vajro P. Oxidative stress in non-alcoholic fatty liver disease. an updated mini review. Front Med (Lausanne). 2021;8:595371. 33718398 10.3389/fmed.2021.595371 PMC7952971

[37] Clifford T Acton JP Cocksedge SP Davies KAB Bailey SJ. The effect of dietary phytochemicals on nuclear factor erythroid 2-related factor 2 (Nrf2) activation: a systematic review of human intervention trials. Mol Biol Rep. 2021;48:1745–61. 33515348 10.1007/s11033-020-06041-x PMC7925463

[38] Sakuma T Nakamura M Chiba T. A diet-induced murine model for non-alcoholic fatty liver disease with obesity and insulin resistance that rapidly develops steatohepatitis and fibrosis. Lab Invest. 2022;102:1150–7. 35643859 10.1038/s41374-022-00807-6

[39] Matsuda M Shimomura I. Roles of adiponectin and oxidative stress in obesity-associated metabolic and cardiovascular diseases. Rev Endocr Metab Disord. 2014;15:1–10. 24026768 10.1007/s11154-013-9271-7

[40] Nigro E Scudiero O Monaco ML. New insight into adiponectin role in obesity and obesity-related diseases. Biomed Res Int. 2014;2014:658913. 25110685 10.1155/2014/658913 PMC4109424

[41] Takahashi E Okumura A Unoki-Kubota H Hirano H Kasuga M Kaburagi Y. Differential proteome analysis of serum proteins associated with the development of type 2 diabetes mellitus in the KK-A(y) mouse model using the iTRAQ technique. J Proteomics. 2013;84:40–51. 23545169 10.1016/j.jprot.2013.03.014

